# Re-validation and update of an extended-specificity multiplex assay for detection of *Streptococcus pneumoniae* capsular serotype/serogroup-specific antigen and cell-wall polysaccharide in urine specimens

**DOI:** 10.1099/acmi.0.000094

**Published:** 2020-01-28

**Authors:** Seyi D. Eletu, Carmen L. Sheppard, Samuel Rose, Kenneth Smith, Nick Andrews, Wei Shen Lim, David J. Litt, Norman K. Fry

**Affiliations:** ^1^​ Vaccine Preventable Bacteria Section, Public Health England – National Infection Service, Colindale Avenue, London, NW9 5EQ, UK; ^2^​ Oklahoma Medical Research Foundation, 825 NE 13th Street, Oklahoma City, OK 73104, USA; ^3^​ Statistics, Modelling and Economics Department, Public Health England – National Infection Service, Colindale Avenue, London, NW9 5EQ, UK; ^4^​ Department of Respiratory Medicine, Nottingham University Hospitals NHS Trust, Nottingham, UK; ^5^​ Immunisation and Countermeasures Division, Public Health England – National Infection Service, Colindale Avenue, London, NW9 5EQ, UK

**Keywords:** diagnostics, monoclonal antibodies, pneumococcus, *Streptococcus pneumoniae*, surveillance studies

## Abstract

National surveillance of pneumococcal disease at the serotype level is essential to assess the effectiveness of vaccination programmes. We previously developed a highly sensitive extended-specificity multiplex immunoassay for detection of *Streptococcus pneumoniae* serotype-specific antigen in urine in the absence of isolates. The assay uses human mAbs that detect the 24 pneumococcal serotype/groups targeted by the pneumococcal conjugate vaccines (PCVs) and pneumococcal polysaccharide vaccine (PPV-23) plus some cross-reactive types and the pneumococcal cell-wall polysaccharide. However, the previous assay had some limitations, namely the reduced specificity of the serotype 7F, 20 and 22F assays, for which non-specific binding in urine samples was observed. Here we report on the further development and re-validation of a new version of the assay (version 2.1), which offers improved sensitivity towards serotypes 7F, 18C and 19F and increased specificity for serotypes 7F, 20 and 22F by replacement of some of the antibody clones with new clones. Using a panel of urine specimens from patients diagnosed with community-acquired pneumonia or pneumococcal disease, the overall clinical sensitivity of this version of the assay based on isolation of *S. pneumoniae* from a normally sterile site is 94.3 % and the clinical specificity is 93.6 %, in comparison with clinical sensitivity and specificity values of 96.2 % and 89.9 % in the previous assay.

## Introduction

*Streptococcus pneumoniae* can be classified into serotypes according to the structure of its cell-wall polysaccharide (CWP) capsule. The total number of serotypes described depends on the criteria and methodology used. Using commercial polyclonal typing sera, 92 are currently described by SSI Diagnostica [1], with a further two (35D and 7D) distinguishable using reactions to available typing sera [2, 3]. Characterization using mAbs [4] and genotyping of the operon encoding capsule synthesis genes has also revealed a number of additional subtypes and variants [5, 6]. This number is likely to increase as more whole genome sequence (WGS) data become available and analysis of the capsular polysaccharide (*cps*) loci is routinely performed. The introduction of the PCV-7 pneumococcal conjugate vaccine (PCV) in 2000 and later the PCV-10 and PCV-13 vaccines in 2010 has helped to reduce the overall incidence of pneumococcal disease (PD) caused by some of the most prevalent serotypes associated with disease and antibiotic resistance at the time of their development [7–9]. However, vaccine-associated serotypes 3 and 19A continue to cause significant numbers of pneumococcal infections whilst previously less common non-vaccine serotypes are starting to increase in prevalence [7–13]. It is therefore important to perform surveillance of PD including serotyping to continually assess the effectiveness of pneumococcal vaccination programmes taking place in countries around the world. Detection and serotyping of non-culturable pneumococci is a valuable tool in the surveillance and treatment of PD. This is particularly true in non-invasive PDs such as pneumonia, where it is reported that, despite being the most common cause of community-acquired pneumonia [14], pneumococci are only isolated from blood culture in less than 25 % of adult pneumococcal pneumonia cases [15, 16].

We previously described the development of an extended-specificity multiplex immunoassay for detection of *S. pneumoniae* serotype-specific antigen in urine using fully human, full-length pneumococcal polysaccharide mAbs (urinary antigen detection, UAD assay) [17, 18]. Although the UAD assay is highly sensitive, the individual assays to detect serotype 7F, 20 and 22F demonstrated poor specificity as a result of non-specific binding to (an) unidentified substance(s) present in some urine samples. We present the development and validation of a new version of the assay (v.2.1) in order to improve the sensitivity and specificity for some of these target serotypes by the inclusion of new mAb clones.

## Methods

### Bacterial strains

The same bacterial isolates and control strains used to validate the previous version of the UAD assay (v.2.0) [17] were used for v.2.1 together with an additional eight clinical *Streptococcus oralis* and eight *Streptococcus parasanguinis* isolates obtained from the Respiratory and Vaccine Preventable Bacteria Reference Unit (RVPBRU), Public Health England (PHE) National Infection Service, Colindale, UK. This made a total of 13 non-pneumococcal streptococcal species (*n*=58 strains), and 111 strains of various non-streptococci bacteria associated with respiratory infections or the urogenital tract, comprising 21 genera and 59 distinct species. Pure cultures of each isolate were suspended in PBS. Bacterial suspensions of >1.2×10^9^ c.f.u. ml^–1^ were heat-killed or treated with 2 % formalin and used to spike 25 mM HEPES (Sigma) buffered pneumococcal antigen-negative urine samples as previously described [17].

### Clinical specimens

In total, 1995 urine samples were obtained from patients diagnosed with community-acquired pneumonia (CAP) upon admission to the Nottingham University NHS Trust hospitals in the UK between 2008 and 2018. Diagnosis of CAP was defined in those patients admitted to hospital with the presence of new or progressive infiltrates on chest radiograph and at least one symptom of acute lower respiratory tract infection, such as cough, fever, dyspnoea, sputum or pleuritic chest pain. These urine samples were frozen at −80 °C prior to transportation to PHE Colindale for testing with the UAD assay. On arrival at Colindale the urine specimens were held at ≤−70 °C for long-term storage and refrigerated at 2–8°C prior to testing. A further panel of 42 urine samples from patients with suspected PD based on clinical symptoms, contact links with confirmed outbreak cases and/or a positive BinaxNOW *S. pneumoniae* test (Alere) were obtained from RVPBRU at PHE Colindale. The RVPBRU urine samples were stored at 2–8 °C.

### Multiplex serotype/serogroup-specific antigen detection assay

The UAD assay was performed as previously described [17] with the exception that the assay diluent no longer contained the negative human reference serum and comprised 2 % (w/v) BSA (Sigma) diluted in PBS at pH 7.4 (Sigma, P3813). The serotype 7F, 18C, 19F, 20 and 22F targeting clones used in version 2.0 of the assay were replaced with new clones. The antibody clones used in both versions of the assay are listed in Table 1 alongside the spectrally distinct Luminex MagPlex bead regions to which they were coupled.

**Table 1.** The human mAb clones used in the previous and current version of the pneumococcal serotype antigen detection assay

Stated serotype specificity	MagPlex bead region	Clone name	
		BioPlex assay v2.0	BioPlex assay v2.1
**1**	77	pn082p5B06	pn082p5B06
**2**	19	pn201p3C02	pn201p3C02
**3**	39	pn201p6E03	pn201p6E03
**4**	65	pn082p3C05	pn082p3C05
**5**	28	pn225p1A02	pn225p1A02
**6A**	13	pn134p1D04	pn134p1D04
**6B**	25	pn201p4G01	pn201p4G01
**7F**	42	pn132p8C04	**pv112Lp8F02**
**8**	46	pn201p4C01	pn201p4C01
**9N**	34	pn201p2D02	pn201p2D02
**9V**	38	pn082p4D06	pn082p4D06
**10A**	29	pn219p4B04	pn219p4B04
**11A**	57	pn082p8F05	pn082p8F05
**12F**	54	pn134p3A05	pn134p3A05
**14**	43	pn225p1A03	pn225p1A03
**15B**	61	pn225p1G05	pn225p1G05
**17F**	66	pn219p1D03	pn219p1D03
**18C**	27	pn082p3G05	**pn132p7A01**
**19A**	35	pn219p4A05	pn219p4A05
**19F**	59	pn132p7B06	**pn132p1E03**
**20**	52	pn219p2D06	**pn134p8B03**
**22F**	26	pn201p3C03	**pn219p5B01**
**23F**	18	pn132p2C05	pn132p2C05
**33F**	63	pn219p1C01	pn219p1C01
CWP*	73	pn082p3C04	pn082p3C04

*CWP refers to the cell-wall polysaccharide antigen target.

### Repeatability

To test the repeatability of the UAD assay, three standard curves, consisting of a mixture of purified capsular polysaccharide (American Type Culture Collection, and SSI Diagnostica) for each of the 25 antibody targets at individual concentrations of 10, 3, 1, 0.3, 0.1, 0.03, 0.01, 0.003, 0.001 and 0.0003 ng ml^−1^ were tested over 5 days, giving 15 results in total. A pneumococcal antigen-negative control urine and 21 additional urine samples were included in the repeatability runs. Fifteen of these were clinical samples obtained between 2008 and 2013, from patients with culture-confirmed or suspected PD based on clinical symptoms, contact links and/or BinaxNOW *S. pneumoniae* antigen results as previously described. One sample was a urine specimen donated from a healthy donor which tested BinaxNOW *S. pneumoniae* antigen-negative and the remaining five samples consisted of BinaxNOW antigen-negative urine specimens that were spiked with purified polysaccharide antigens to produce a final concentration just above the estimated sensitivity cut-off as follows: serotype 2 polysaccharide at 0.04 ng ml^−1^, 17F at 0.1 ng ml^−1^, 20 at 0.04 ng ml^−1^, 22F at 0.04 ng ml^−1^, and a combination of serotypes 23F and 33F at 0.04 ng ml^−1^. Each urine sample was tested in triplicate across the five days.

### Interpretation of results

The test sample to negative control (t/n) ratio of the median fluorescence intensity (FI) reported by the BioPlex was calculated and used to determine the presence of pneumococcal antigen within a sample. For each sample these t/n ratios were normalized using a method similar to that described by Sheppard *et al*. [19] to account for any samples producing elevated background signals when compared to the negative control. This normalization involves dividing the t/n ratios by the 80^th^ percentile of the t/n ratios across the serotypes for that sample and therefore assumes that there are no more than four positive results (excluding the CWP) for each sample. A second normalization step was then performed by dividing each of the ratios reported for the individual serotype assays by the median of the normalized t/n ratios for all samples in that same serotype assay. This second normalization can only be used in cases where the majority of samples are negative and therefore the median would represent a negative result. Any results above or equal to the normalized t/n ratio of 2.0 were considered positive.

## Results

### Examination of the positivity cut-off

The UAD v.2.0 assay uses a normalized t/n ratio of 2.5 as the positivity cut-off, calculated as previously [17]. Analysis of the FI data produced from the repeatability runs in UAD v.2.1 revealed that an equivalent t/n ratio of 1.8 is ≥5 sd above the mean FI of the negative controls in all 25 serotype assays and therefore, based on these data, would indicate >99.9 % certainty of positivity compared to the negative control urine sample (Table 2). Additionally, a normalized t/n ratio of 1.8 is ≥4 sd above the average of the normalized ratios for the negative (t/n<2.0) clinical samples (*n*=1194) tested in all 25 assays. This demonstrates that results above or equal to a normalized t/n ratio of 2.0 are discernibly above the negative control and, consequently, a normalized t/n ratio of 2.0 was set as the general positivity cut-off for the analysis of the urine results.

**Table 2.** Normalized ratios of the negative control urine samples (*n*=15) and estimated cut-offs calculated as 5 sd greater than the mean of the normalized negative control ratios

Stated serotype specificity (bead region)	Mean normalized ratio	sd	Mean ratio plus 5 sd
1 (77)	1.1	0.04	1.3
2 (19)	1.1	0.08	1.5
3 (39)	1.5	0.06	1.8
4 (65)	1.1	0.06	1.4
5 (28)	1.1	0.11	1.65
6A (13)	1.1	0.08	1.5
6B (25)	1.1	0.07	1.45
7F (42)	1.1	0.04	1.3
8 (46)	1.2	0.09	1.65
9 N (34)	1.1	0.03	1.25
9V (38)	1.1	0.04	1.3
10A (29)	1.1	0.04	1.3
11A (57)	1.1	0.05	1.35
12F (54)	1.1	0.07	1.45
14 (43)	1.1	0.02	1.2
15B (61)	1.1	0.07	1.45
17F (66)	1.2	0.06	1.5
18C (27)	1.1	0.05	1.35
19A (35)	1.1	0.03	1.25
19F (59)	1.0	0.06	1.3
20 (52)	1.1	0.02	1.2
22F (26)	1.1	0.05	1.35
23F (18)	1.1	0.09	1.55
33F (63)	0.7	0.02	0.8
CWP* (73)	1.0	0.06	1.3

*CWP refers to the cell-wall polysaccharide antigen target.

### Analytical sensitivity

The limit of detection for the purified capsular polysaccharides for each assay was estimated using the standard curve dilutions as 3 sd above the mean FI of the negative controls in the repeatability runs. Using the value at 3 sd above the mean of each serotype assay negative control as a cut-off, it was estimated that serotypes 1, 2, 3, 4, 5, 8, 9V, 11A, 14, 18C and 20 can be detected at or below 0.3 pg purified polysaccharide per ml (0.3 pg ml^−1^), serotypes 6A, 10A, 15B, 19A, 19F and 22F can be detected at concentrations as low as 1 pg ml^−1^, serotypes 7F, 12F and 33F at concentrations as low as 3 pg ml^−1^, serotypes 6B, 9N and 23F as low as 10 pg ml^−1^, and serotype 17F can be detected as low as 30 pg ml^−1^.

### Analytical specificity

Similar cross-reactions to those observed in v.2.0 of the UAD were seen in v.2.1 [17], with the exceptions that the new serotype 18C targeting mAb clone no longer cross-reacted with serotypes 35C and 42, the new clone targeting serotype 19F cross-reacted with all the serogroup 19 serotypes, and the clone targeting 22F no longer cross-reacted with serotypes 22A and 43 but did cross-react with serotypes 6C and 6D if present at high concentrations. The clone targeting serotype 22F also occasionally cross-reacted with serotype 20. The false-positive reporting observed for the previous clones targeting serotypes 7F, 20 and 22F as a result of non-specific binding with an unknown substance in some urine samples [17] was not observed with the new clones. Taking the cross-reactions into account, positive serotype 6A, 6B, 7F, 9V, 10A, 11A, 12F, 15B, 18C, 19F, 23F and 33F results for v.2.1 of the UAD would be reported as positive for serotype antigens 6A/C, 6B/D, 7A/F, 9A/V, Group 10/33C/39, 11A/C/E/Group 16, 12F/44/22A, Group 15, 18B/C/F (or Group 18), Group 19, 23F/Group 32 and 33A/B/D/F, respectively. Table 3 lists the cross-reactions observed for each of the clones.

**Table 3.** The human mAbs that cross-react with non-targeted pneumococcal serotypes

Human mAb target (bead region)	Cross-reaction							
**6A (13**)	6B*	6C	6D					
**6B (25**)	6A*	6D						
**7F (42**)	7A							
**9N (34**)	9L	**18A†**	**43**	**47A**				
**9V (38**)	9A	9L						
**10A (29**)	10B	10C	10F	**33C**	**39**			
**11A (57**)	11C	11D	11E	**16A**	**16F**	(**18A**)‡	(**18B**)	(**18C**)
**12F (54**)	12B	**22A**	**44**					
**14** (**43**)	**9L**	9N*						
**15B (61**)	15A	15C	15F	**11D**	**12B**	**18A**		
**18C (27**)	18A	18B	18F					
**19A (35**)	19F*	**9A**	**9L**	9V*	(**18B**)	18C*		
**19F (59**)	19A*	19B	19C					
**22F (26**)	(**6C**)	(**6D**)	(20)*					
**23F (18**)	**32A**	**32F**						
**33F (63**)	33A	33B	33D					

*Cross-reaction with polysaccharide antigen detected in the 25plex assay.

†Serotypes in bold indicate a cross-reaction with a different serogroup.

‡Serotypes in parentheses indicate a possible reaction that is not always seen.

The analytical specificity of the UAD v.2.1 assay to non-pneumococcal antigens was also similar to that of the UAD v.2.0 assay [17] with the exception of the type strain for *S. parasanguinis* (ATCC 15912^T^) which tested positive for the Group 19 antigen (as detected by the 19F target clone) but still negative for the CWP antigen. The assay was used to test a further eight *S. parasanguinis* and eight *S. oralis* clinical isolates. Of these, two out of eight of the *S. oralis* strains gave a positive result for serotype 10A/33C/39, one gave a positive serogroup 19 result and one was positive for the CWP antigen only. The remaining *S. oralis* and the *S. parasanguinis* did not produce a positive result. Therefore, of the 59 non-pneumococcal species, 10 *Streptococcus agalactiae* serotypes and seven *Haemophilus influenzae* serotypes tested with the UAD v.2.1 assay, 73 were negative for serotype antigen and 72 were CWP-negative, resulting in analytical specificities of 96.1 % [95 % confidence interval (CI): 88.9–99.2] and 94.7 % (95 % CI: 87.1–98.6) against non-pneumococcal bacteria for the serotype and CWP assays respectively. None of the non-pneumococcal bacteria tested gave positive results for both a serotype and the CWP antigens.

### Clinical testing

In total, 2037 urine samples were tested with the UAD v.2.1 assay, of which 441 (21.6 %) were obtained from patients with PD as defined by a positive culture isolate from blood or bronchoalveolar lavage (BAL) and/or a positive BinaxNOW *S. pneumoniae* antigen test result (Table 4). The UAD assay identified 398 (90.2 %) of these 441 urine samples as positive for pneumococcal antigen and, of these, 313 were serotyped. Of the 1596 urine samples negative for pneumococci by the BinaxNOW test and with no associated pneumococcal blood or BAL culture, our assay detected a further 456 (22.4 %) as positive for pneumococcal antigen, 114 of which were positive for both the serotype and the CWP antigens. Therefore, a total 854 (41.9 %) of the tested urine samples were positive for pneumococcal antigen with the UAD v.2.1 assay, 719 (35.3 %) of which were serotyped. Multiple serotypes were detected in 107 (5.3 %) of the 2037 urine samples.

**Table 4.** Comparison of *Streptococcus pneumoniae* sterile site culture and BinaxNOW diagnostic methods with the multiplex serotype-specific antigen detection assay (UAD) on samples from patients with community acquired pneumonia (CAP) (*n*=1995) or suspected pneumococcal disease (PD) (*n*=42)

CAP and PD cases	No. of cases	No. positive by UAD (%)	No. serotyped by UAD (%)
* S. pneumoniae* culture-positive	88	83 (94.32)	75 (91.46)
Culture-positive with UAD serotype	82	77 (93.90)	75 (91.46)
Culture-positive with non-UAD serotype	6	6 (100)	0 (0)
BinaxNOW-positive	412	374 (90.78)	289 (70.15)
* S. pneumoniae* culture and/or BinaxNOW-positive	441	398 (90.25)	313 (70.98)
**Total no. of urine samples**	**2037**	**854** (**41.9**)	**719** (**35.3**)

Pneumococci were isolated and serotyped from blood culture or BAL specimens of 88 of the patients from whom urine samples were tested. When compared, the UAD v.2.1 assay was able to detect pneumococcal antigen in 83 (94.3 %) of the urine samples from these patients and generate serotype information from 75 of the 83 urine samples; 74 of these serotypes matched the serotype reported from the paired culture isolate, whilst six of the culture-identified isolates were serotypes which are not currently typeable with the UAD v.2.1 assay [serotypes 23A (*n*=1), 24F (*n*=2), 31 (*n*=1) and 35B (*n*=2)]. Nevertheless, the CWP was detected in all six of these paired non-typeable urine samples. The single discrepant result was due to a urine sample with a paired isolate serotyped as 16F. The 16F antigen is detectable in the UAD assay due to a cross-reaction with the 11A targeting clone (Table 3) and in the case of this particular urine sample, on initial testing an 11A/C/E or serogroup 16 antigen was detected at the positivity cut-off along with the serotype 5 antigen. However, on repeat testing the 11A/C/E or serogroup 16 antigen was detected below the positivity cut-off whilst the serotype 5 antigen was detected just above the cut-off, and therefore the sample was reported as positive for the serotype 5 antigen only. The BinaxNOW pneumococcal test can detect pneumococcal cell-wall C polysaccharide in urine and cerebrospinal fluid (CSF) samples with reported sensitivities of 74–90 % and specificities of 71–97 % [20–23], although sensitivities may vary in relation to the serotype [23]. In total, 412 urine samples tested positive for the pneumococcal antigen with the BinaxNOW test and 374 (90.8 %) of these were positive for a pneumococcal serotype and/or CWP antigen with the BioPlex assay.

Using the method described by Huijts *et al*. [24], which calculates the specificity of a pneumococcal serotype-specific urine antigen test (UAT) using urine samples from CAP patients by comparing the number of UAT-negative samples with the number of ‘true negatives’, defined as CAP cases with bacteraemia caused by another pathogen or CAP cases with only a positive Legionella UAT, 94 of the urine samples tested with our UAD v.2.1 assay would be described as true negatives. Of these, 88 samples produced a negative result when tested in our assay, resulting in a clinical specificity of 93.6 % (95 % CI: 86.6–97.6). Forty-eight of the tested urine samples were from CAP patients defined as ‘true negative’ due to bacteraemia caused by a pathogen other than *S. pneumoniae *, and of these 42 were negative when tested with our UAD v.2.1 assay, with a resulting specificity of 87.5 % (95 % CI: 74.8–95.2).

## Discussion

Pneumococci continue to be a major cause of morbidity and mortality worldwide in the era of pneumococcal conjugate and polysaccharide vaccines. To assist with the detection and surveillance of PD, a UAD assay capable of detecting 24 pneumococcal serotype/groups plus some cross-reactive types and the pneumococcal CWP in the urine samples of patients with non-invasive diseases such as pneumonia was developed [17]. The assay uses 25 fully human, full-length pneumococcal polysaccharide mAb clones that target the pneumococcal serotypes/groups. Each antibody clone has different affinities and avidities for its target and this is reflected in the different sensitivities observed towards the various serotypes. We have now improved upon the previously published version of the assay by replacing some of the mAb clones to increase the sensitivity of the assay towards serotypes 7F, 18C and 19F. The replacement 19F targeting clone, however, is no longer specific for serotype 19F and instead targets all the serogroup 19 serotypes. The cross-reactions observed with the 19F targeting clone, as well as some of the other cross-reactions described in Table 3 may be due to specific similarities in the polysaccharide structures between the serotypes, with certain clones targeting these in-common structures. Indeed, this is often the case for serotypes within the same serogroup [25]. The specificity of the assay for serotypes 7F, 20 and 22F was also improved as previous clones were observed to report false-positive results in some urine samples due to non-specific binding, meaning that the UAD v.2.1 assay can now detect serotypes 7F, 20 and 22F at lower concentrations with more certainty. The negative human reference serum was included in the assay diluent of UAD v.2.0 to reduce any non-specific binding that may occur when testing clinical samples in immunoassays using human mAbs. We decided to investigate the possibility of removing the negative human reference serum from the assay diluent as this represented an additional variable for which batch to batch variation would be difficult to control. Experiments after removal from the assay demonstrated that non-specific binding is not a significant problem with UAD v.2.1 although there were slight increases in the background FI signals of some individual serotype assays (data not shown), which may have led to the observed differences in the limit of detection for some of the individual serotype assays. However, despite this the analytical sensitivity of each individual serotype assay remains high. Overall, the clinical sensitivity of the UAD assay increased from 93.2 % with v.2.0 to 94.3 % with v.2.1 when compared to detection by isolation of *S. pneumoniae* from a normally sterile site. In addition, the clinical specificity of the UAD has increased from 89.9 % to 93.6 % with v.2.1.

As with the previous version of the assay, we observed cross-reactivity of some mAb clones to other *mitis* group streptococci. This may be due to these non-pneumococcal streptococci carrying similar genes to those found in the pneumococcal capsular operon, which express similar polysaccharides to those found in pneumococci, including the pneumococcal cell wall [26–31]. However, these streptococci are associated far less with CAP or urine infections and are therefore less likely to be detected or isolated from the urine specimens of patients with clinical disease [32–34]. Additionally, the high sensitivity of the UAD assay means that it may be possible to detect pneumococcal antigen in urine samples of individuals who are carrying pneumococci due to colonization of their nasopharynx. Indeed, BinaxNOW-positive results have been reported in healthy children who have been colonized with pneumococci [35–38]. The highest carriage rates have been observed in children and individuals residing in lower income countries, where studies have estimated rates to be between 6.4 and 93.4 % [13, 39–45]; by contrast, in England, carriage in adults is predicted to be much lower, at approximately 2.8 % [45]. It is therefore possible that a small proportion of positive results observed with our UAD assay are due to other *mitis* group streptococci or pneumococcal carriage and this may also contribute to some of the results with multiple serotypes. Consequently, it is important that results of the UAD assay are interpreted in the context of clinical presentation and other laboratory test results. Nevertheless, the UAD assay is able to provide serotype information from non-invasive samples and identify patients infected with/carrying multiple serotypes, which is difficult to perform using culture-based methods. Furthermore, when used to test urine samples from CAP and suspected PD patients, this new version of the UAD assay was able to identify an additional 22.4 % of urine samples from patients originally described as negative for pneumococcal CAP or PD using culture from normally sterile sites and BinaxNOW *S. pneumoniae* antigen methods of detection. This UAD assay offers the capacity for further development with the introduction of novel anti-pneumococcal serotype-specific mAbs. Additionally, this assay could be used to identify pneumococcal antigens in other clinical samples such as blood, CSF and pleural fluid (data not shown).
